# Minimal residual disease in pediatric ALL

**DOI:** 10.18632/oncotarget.20856

**Published:** 2017-09-13

**Authors:** Ching-Hon Pui, Dario Campana

**Affiliations:** Ching-Hon Pui: Departments of Oncology and Pathology, St. Jude Children’s Research Hospital, Memphis, TN, USA

**Keywords:** ETV6-RUNX1, hyperdiploid >50, early T-cell precursor ALL, genotype, deep sequencing

In pediatric acute lymphoblastic leukemia (ALL), 5-year overall survival rates with contemporary risk-directed treatment exceed 90% [1]. To further improve these results and diminish the toxicity associated with intensive therapy, current efforts focus on expanding the application of immunotherapy, identifying targetable genetic abnormalities, and improving the precision of risk assessment. To this end, minimal residual disease (MRD) level determined during the early phases of treatment (remission induction or consolidation) has been an independent prognostic factor in all reported clinical trials for pediatric and adult ALL. Importantly, the application of MRD, measured by flow cytometry and/or molecular methods, to guide treatment intensity has contributed to improve outcomes. Thus, Total Therapy Study XV, the first clinical trial featuring MRD-based response-directed therapy, resulted in a 5-year overall survival of 94% [1], with excellent results extending to subgroups of ALL historically associated with an unfavorable outcome, such as hypodiploid (74%) or Philadelphia chromosome-like ALL (93%) [2]. Randomized clinical trials also demonstrated the feasibility of reducing therapy to avert toxicity while maintaining excellent outcome in patients with clinical standard- or intermediate-risk ALL and undetectable MRD (i.e., <0.01%) at the end of remission induction [3]. However, leukemia relapse in 4% to 6% of such patients [1-4] cautioned that such approach must be implemented within a refined risk stratification schema and with adequate treatment intensity.

Proper interpretation of MRD results must consider other factors, such as timing of measurement, leukemia subtype, and efficacy of treatment [2]. Depending on protocol, MRD has been studied during remission induction therapy on day 8 and day 15/19, upon completion of remission induction (day 29, 33 or 42), and at the end of consolidation treatment (day 78, week 14, or 15) [2]. In general, early measurements are useful to identify patients with drug-sensitive leukemia, who can attain MRD-negative status after one to two weeks of remission induction with 3 or 4 chemotherapeutic drugs, and might benefit from an overall reduction in the intensity and/or duration of post-remission treatment. Data from the Total Therapy Study XV indicated that the most suitable candidates for such reduction are patients with favorable leukemia genotypes, such as *ETV6-RUNX1* fusion or hyperdiploidy >50 [4]. Among patients with negative MRD at day 19 of remission induction, those with these genetic features had the lowest 10-year cumulative risk of relapse (1.9% and 3.8%, respectively), while other NCI standard-risk patients had a cumulative risk of 9.5% [4]. This difference might help explaining the results of the AIEOP-BFM-ALL 2000 study, in which NCI standard-risk patients with negative MRD on days 33 and 78 were randomized to receive standard (protocol II) or abbreviated and less intensive delayed intensification treatment (protocol III): the latter approach yielded higher cumulative risk of relapse (6.3% vs. 3.2% at 4 years) and poorer survival (96.1% vs. 98% at 8 years) [5]. Conceivably, day 33 MRD measurements and the NCI standard-risk classification (based on only age and leukocyte) misclassified some cases as low-risk. Indeed, results of Total Therapy Study XV showed that MRD at the end of remission induction (day 46) was, by itself, insufficient to identify very low-risk patients; 10-year cumulative risk of relapse was 4.7% for *ETV6-RUNX1* ALL, 5.6% for other NCI standard-risk B-ALL, and 6.3% for hyperdiploid >50 ALL, increasing to 12.7% for NCI high-risk B-ALL and 15.5% for T-cell ALL [4]. In the UKALL 2003 study, the cumulative risk of relapse for patients with intrachromosomal amplification of chromosome 21 and negative MRD at the end of induction (day 28) was as high as 28%, despite intensive post-remission therapy [6]. Hence, timing of the MRD measurement and leukemia subtype highly impact the prognostic significance of MRD. Novel MRD methods that can reliably detect MRD at or below the 0.001% level [2] might help identifying patients with highly chemo-sensitive and, hence, highly curable leukemia, but supporting correlative data is still lacking.

High levels of MRD (e.g., ≥1%) at the end of remission induction (after exposure to as many as 7 or more drugs), and/or persistent MRD after consolidation treatment predict a dismal prognosis if treatment consists of chemotherapy alone, and have been used as an indication for allogeneic hematopoietic cell transplantation [2,4,7]. Recent studies suggested that a substantial proportion of hyperdiploid >50 or early T-cell precursor ALL patients with poor response to remission induction might be cured with effective post-remission treatment (consolidation therapy with high-dose methotrexate and mercaptopurine for the former, and consolidation phase IB therapy with cyclophosphamide, mercaptopurine and cytarabine for the latter patients) [7,8]. In this regard, we have shown that sequential MRD measurements beyond the remission induction phase were informative if patients had detectable MRD at the end of remission induction; chemotherapy alone could cure patients with decreasing MRD post-remission while those with increasing MRD required alternative therapy [1]. By contrast, routine sequential monitoring of MRD in patients who are MRD-negative at the end of remission induction is probably unnecessary. In our experience, the vast majority of subsequent tests will be negative, and there is no evidence that modifying the treatment plan as soon as MRD re-emerges will improve outcome in pediatric ALL [1].

With the expanding array of novel therapies for ALL, the applications of MRD tests are extending beyond traditional prognostication, and providing clear eligibility criteria and response parameters that should facilitate the rapid identification of the most effective agents to improve outcome.
